# Mural Nodules of Clear Cell Carcinoma in a Mucinous Borderline Tumor of the Ovary: A Case Report

**DOI:** 10.4061/2010/438534

**Published:** 2010-04-13

**Authors:** Daniela S. Allende, Richard D. Drake, Longwen Chen

**Affiliations:** ^1^Department of Anatomic Pathology, The Cleveland Clinic, Cleveland, OH 44195, USA; ^2^Department of Obstetrics and Gynecology, The Cleveland Clinic, Cleveland, OH 44195, USA

## Abstract

Mural nodules of ovarian mucinous borderline tumors are rare. In this study, we report a case of mural nodules of clear cell carcinoma in an intestinal type mucinous borderline tumor of the ovary. The patient was a 54-years-old woman presented with back and pelvic pain for 3 months. A right-sided multiloculated ovarian mass approximately 20 cm was identified on the CT scan. CA-125 was moderately elevated. She underwent total abdominal hysterectomy with bilateral salpingo-oophorectomy and bilateral pelvic and para-aortic lymphadenectomy. Grossly, the right ovarian mass showed a multiloculated cystic mass with mucinous fluid. There were papillations in the internal surface and two mural nodules were seen. Microscopic examination revealed that the cystic mass was an intestinal type borderline mucinous tumor. The mural nodules showed a classic histology of clear cell carcinoma with tubulocystic and papillary growth patterns. This is an extremely rare case of mural nodules of clear cell carcinoma arising in a mucinous borderline tumor.

## 1. Introduction

Mucinous ovarian tumors were first described in 1809 by Ephraim McDowell [1]. Nowadays, mucinous ovarian tumors represent up to 15% of all ovarian tumors. In the current WHO classifications of ovarian neoplasms, mucinous tumors are classified as surface epithelial-stromal tumors. Primary mucinous ovarian tumors are further classified into benign, borderline, or malignant categories depending on their histologic features [2]. Borderline tumors account for 10% of all the mucinous tumors [3]. Mucinous borderline tumors have been further subclassified as intestinal and endocervical types [3]. Intestinal-type mucinous borderline tumors tend to be more heterogeneous in composition and may contain areas of cystadenoma and noninvasive carcinoma [4]. Endocervical-type mucinous borderline tumors are more closely related to borderline serous tumors [4]. On rare occasions, one or more discrete well-circumscribed mural nodules protrude into the lumen of a cystic ovarian epithelial neoplasm; most commonly occur in mucinous tumors of intestinal type [4]. The nodules may be reactive or neoplastic, and some have mixtures of both types. Mural nodules with foci of anaplastic carcinoma, sarcoma, sarcoma-like, and carcinosarcoma-like nodules have been reported in the literature [5–7].

We hereby report a case of mural nodules of clear cell carcinoma in an intestinal-type borderline mucinous ovarian tumor. Although clear cell carcinomas have been found in association with mucinous cystadenomas and endometriosis in the past [8–10], to our knowledge, this is the first report of mural nodules of clear cell carcinoma within a mucinous borderline tumor in the English literature.

## 2. Case Report

The patient was a 54-years-old G2P2 caucasian woman with a past medical history of hypertension, hyperlipidemia, hernia, obesity and laparoscopic cholecystectomy. She complaint of 3 months of pelvic and back pain. The pain was described as aching, occurring on daily basis and 4/10 in an scale of severity. She also had early satiety, occasional nausea, and abdominal distention for the past 6 months. She had vaginal spotting for the last 2 weeks at the moment of presentation.

Physical examination revealed a large pelvic-abdominal mass, approximately 20 cm in greatest dimension. Abdominal-peilvic CT scan revealed a 20 × 10 × 7 cm right multiloculated ovarian mass. CA-125 was 43 U/ml (normal <35 U/mL). The patient was scheduled for surgery. A exploratory laparotomy was performed with frozen section consultation. The intraoperative diagnosis was at least borderline mucinous ovarian neoplasm. A total abdominal hysterectomy, bilateral salpingo-oophorectomy, and bilateral pelvic and aortic lymphadenectomy were performed. Ovarian staging, including multiple biopsies and pelvic washings, was done. 

After surgery, she started 6 cycles of chemotherapy with carboplatin/taxol. At present, patient was on her second cycle without any complication related to the treatment.

## 3. Gross Findings

**Figure 1:**
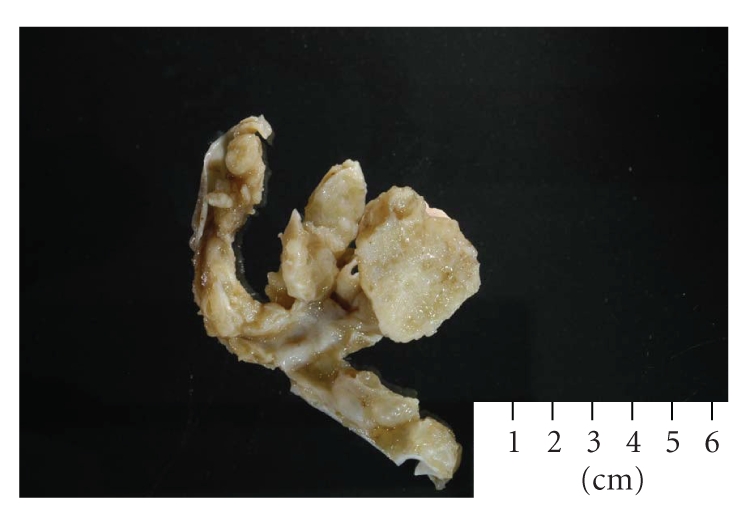
The gross appearance of one of the mural nodules.

The right ovary was enlarged and completely distorted by a mass which measured 25 × 22 × 15 cm and weighted 5.2 kg. The ovary had attached 7 cm in length fallopian tube which was unremarkable. The external surface of the ovary was intact, smooth, and glistening. Upon serial sectioning, the ovarian parenchyma was completely replaced by a multiloculated cystic mass with mucinous fluid inside the cystic spaces. The internal surface of the cyst was mostly smooth with areas of prominent friable papillae. Two solid nodules, representing mural nodules, were identified and measured 5 and 3 cm in greatest dimension (Figure 1). These mural nodules protrude into the lumen of this cystic ovarian neoplasm and are sharply demarcated from the rest of the lesions and showed distinctive characteristics, such as tan discoloration, soft and spongy consistency as well. Focal areas of purulent material were identified within the nodules. The entire tumor was extensively sample in 26 blocks of paraffin.

## 4. Microscopic Findings

**Figure 3:**
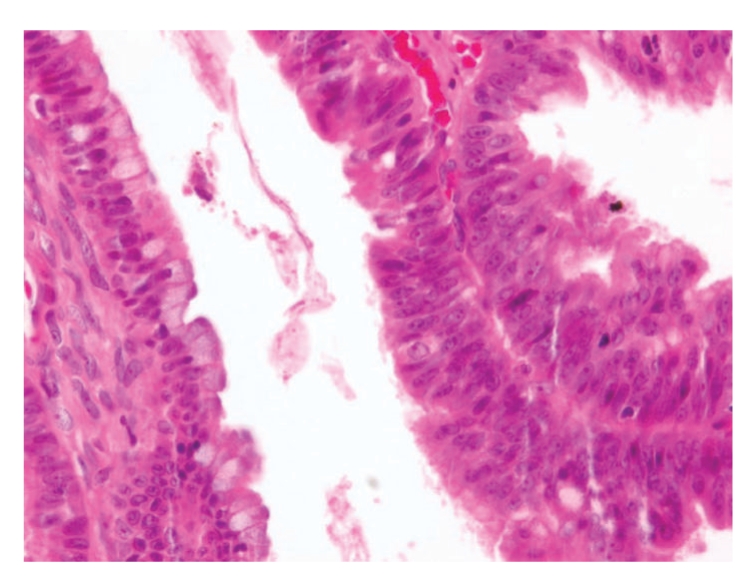
The cells of the intestinal mucious borderline tumor show moderate cytologic atypia, inceased nuclear to cytoplasmic ratio, and visible nucleoli. Goblet cells are present.

The ovarian tumor was composed by multiple cystic spaces and glands with variable size and shapes; some were lined by a single layer of intestinal-type epithelium, resembling mucinous cystadenoma. Multiple delicate papillary infoldings were identified. The cells in those areas were more crowded, stratified in two or more layers with loss of the polarity. Occasional mitotic figures and mild to moderate cellular atypia were seen (Figure 3). Severe cytological atypia was absent. The intervening stroma was unremarkable and areas of invasion were not seen. 

**Figure 4:**
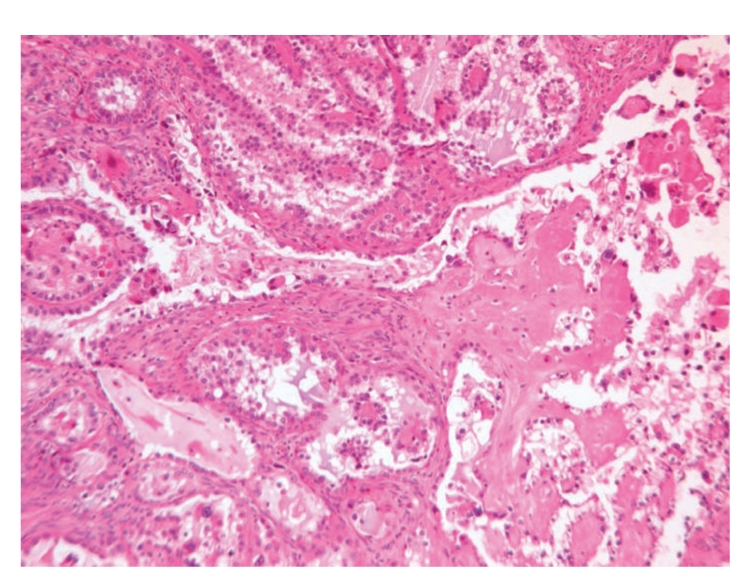
The mural nodule of clear cell carcinoma shows papillary and tubulocystic patterns with prominent stromal hyalinization.

The mural nodules showed abrupt transition from the surrounding mucinous borderline tumor and microscopically displayed a tubulocystic, papillary, and focally solid proliferation of neoplastic polyhedral cells with abundant clear cytoplasm (Figure 4). The nuclei showed moderate to severe cytological atypia with clumped chromatin and prominent nucleoli (Figure 4). The stroma and papillary cores had prominent hyalinization. Evidences of endometriosis or endometrioid carcinoma were not seen.

The tumor was diagnosed as intestinal borderline mucinous tumor with mural nodules of clear cell carcinoma.

## 5. Discussion

Mucinous ovarian tumors are among the most difficult ovarian tumors for surgical pathologist to interprete. In the current WHO classification of ovarian neoplasms, mucinous tumors are classified within the surface epithelial-stromal tumor category [2]. Unlike the serous tumors, intestinal-type mucinous ovarian tumors are frequently heterogeneous in their cell type composition and degree of differentiation. This heterogeneity causes many problems in diagnosis of these tumors [4]. Thus, careful gross examination and widespread sampling is important to identify the most important component related to prognosis, such as invasive carcinoma. In this case, we did very careful gross examination and extensive sampling with more than 20 sections per 20 cm tumor. We grossly identified two mural nodules.

Even though rare, mural nodules have been described in benign, borderline, and malignant mucinous ovarian tumors. The mural nodule could be benign (usually mimicking a malignancy) or malignant in nature [3, 4]. Bague et al. [7] published the largest series of sarcoma-like nodules in mucinous tumors with 10 cases. By the time of their report, only 7 additional cases of this entity were reported. These mural nodules tend to appear in younger patients and they have an excellent prognosis. Several more case reports have described mural nodules of anaplastic carcinomas, carcinomas NOS, and sarcomas in mucinous neoplasms [5, 6]. At least the sarcomatous nodules have been related to older patients. The presence of a malignant mural nodule in a mucinous tumor has serious prognostic implications for the patient depending on the FIGO staging. It is reported that those mural nodules composed of anaplastic carcinoma, sarcoma, or both have a 50% mortality rate [11].

Clear cell carcinoma was first described in 1939 by Schiller [12]. The term of mesonephroma, a misnomer, was introduced because it was regarded as of mesonephric rest derivation. Scully [13] subsequently provided convincing evidence that clear cell carcinoma is of mullerian nature and related to endometrioid carcinoma. This tumor usually occurs in middle-aged patients and has been associated with endometriosis of the ovary. Stage by stage, the prognosis of clear cell carcinoma is similar to that of patients with other epithelial ovarian carcinomas [14].

Dutt and Berney reported for the first time a clear cell carcinoma arising in a mucinous cystadenoma, also with associated endometriosis [8]. This report was controversial and criticized by some authors as misdiagnosed areas of mucinous metaplasia and not a truly mucinous cystadenoma [15]. Later, Uddin and Wani reported two separate cases of clear cell carcinoma arising from a mucinous cystadenoma [9, 10]. Our case is quite different from the above reported cases. First, in our case, the background is an intestinal type mucinous borderline tumor, not benign mucinous cystadenoma. Second, none of above reported cases described the clear cell carcinoma as a mural nodule within the cystic ovarian tumor, although solid areas were present in one of the cases. Soslow mentioned in a review article that clear cell areas are sometimes found in endocervical mucinous borderline tumor [16]. In this case, it is intestinal type mucinous borderline tumor and the clear cell carcinoma is grossly and microscopically distinct from the borderline mucinous tumor. To our knowledge, clear cell carcinoma as a mural nodule in a borderline intestinal-type mucinous tumor has not been previously reported in the English literature.

In our case, the mural nodules showed characteristic features of clear cell carcinoma with tubulocystic and papillary architectures. The neoplastic cells had abundant clear cytoplasm and moderate to severe atypia. The stroma was extensively hyalinized as it is frequently described in this entity. The intestinal type borderline mucinous tumor areas were classical, showing cystic spaces with papillae lined by focally stratified intestinal-type epithelium and without evidence of stromal invasion. The overall gross appearance and microscopic findings in both the borderline tumor and clear cell carcinoma are classic; we feel that immunohistochemical or special studies are not necessary for the diagnosis.

In summary, we have reported an exceedingly rare case of clear cell carcinoma arising in an ovarian mucinous borderline tumor of the intestinal type. Careful gross examination and extensive sampling are crucial for the correct diagnosis. Since malignant neoplasm within mural nodules has a significant impact in the prognosis of the patients, pathologists have to be able to identify these mural nodules accurately.

## Figures and Tables

**Figure 2:**
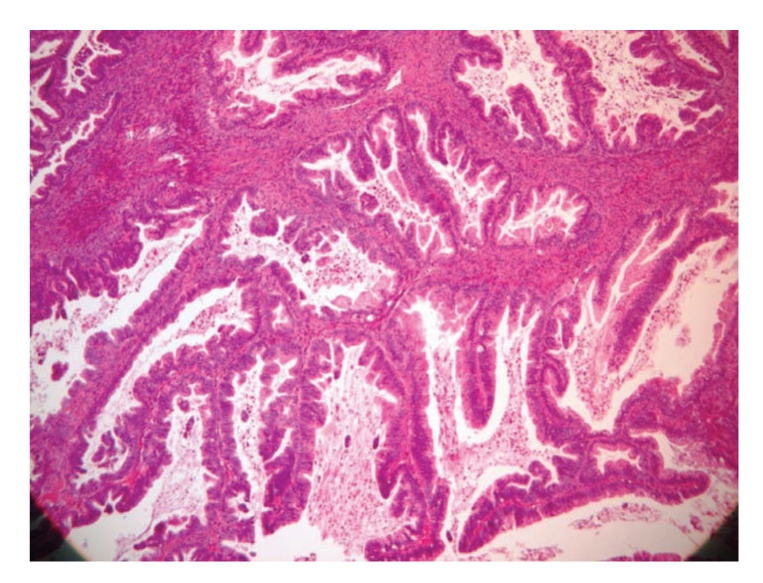
Intestinal mucinous borderline tumor with complex villous architecture.
